# 98. Resurgence of Invasive Group A Streptococcal Disease in Utah Children

**DOI:** 10.1093/ofid/ofad500.014

**Published:** 2023-11-27

**Authors:** Benjamin McMillion, Nicole L Pershing, Hillary Crandall, Kwabena Krow Ampofo, Adam Hersh, Jared Olson, Kelly F Oakeson, Jennifer M Wagner, Erin L Young, Shannon Nielsen, Mandy Dickey, Kelly Huynh, Andrew T Pavia, Anne J Blaschke

**Affiliations:** University of Utah School of Medicine, Salt Lake City, UT; University of Utah School of Medicine, Salt Lake City, UT; University of Utah School of Medicine, Salt Lake City, UT; University of Utah School of Medicine, Salt Lake City, UT; University of Utah, Salt Lake City, UT; Primary Children's Hospital, Salt Lake City, Utah; Utah Department of Health & Human Services / Utah Public Health Laboratory, Taylorsville, Utah; Utah Department of Health and Human Services, Taylorsville, Utah; Department of Health and Human Services, Public Health Laboratory, Salt Lake City, Utah; University of Utah School of Medicine, Salt Lake City, UT; Primary Children's Hospital, Salt Lake City, Utah; Intermountain Health, Salt Lake City, Utah; University of Utah, Salt Lake City, UT; University of Utah School of Medicine, Salt Lake City, UT

## Abstract

**Background:**

*Streptococcus pyogenes* (GAS) is a common cause of non-invasive mucosal infections in childhood as well as life-threatening invasive infections. Since 1997, *emm1* has been the most common *emm* type associated with invasive GAS (iGAS) in the US. Apparent increases in iGAS have been reported in several countries during the winter of 2022-23. We sought to characterize changing clinical and molecular epidemiology of iGAS disease in Utah children before and after the emergence of COVID-19.

**Methods:**

We retrospectively identified children 0-18 years with iGAS treated at Primary Children’s Hospital (Salt Lake City, Utah) from 2018- 2023. Cases were identified through the laboratory database. Electronic health record review confirmed that cases met the CDC definition of iGAS and streptococcal toxic shock syndrome (STSS). We abstracted demographics, disease classification and outcomes (Table). Comparisons were made across years. GAS isolates underwent whole genome sequencing for *emm* type determination.

**Results:**

From 1/2018 to 3/2023, we identified 141 cases of iGAS. Case numbers ranged from 6/yr (2021) to 39 (2019). The highest number of cases by quarter was seen in Q1 2023 (Figure). We observed a marked decrease in iGAS from Q1 2020 through Q3 2022 followed by a steep increase, coinciding with surges in RSV and influenza. Demographics were similar across the study period. 60% of iGAS cases were in males. Complicated pneumonia and head/neck infections predominated. iGAS associated with STSS appeared higher post-pandemic (4/73 [5%] cases in 2018-19 vs. 8/51 [16%] in 2022-23 [OR = 3.2; 95% CI 0.8-15]). The majority of STSS (7/12; 57%) was associated with complicated pneumonia. Clinical outcomes were comparable pre- and post-pandemic. *emm* types were available for 49/51 (96%) of isolates in 2022-23. *emm1* and *emm12* were associated with 67% of iGAS disease (*emm1* n = 16 [33%]; *emm12* n = 17 [35%]).

**Figure:**
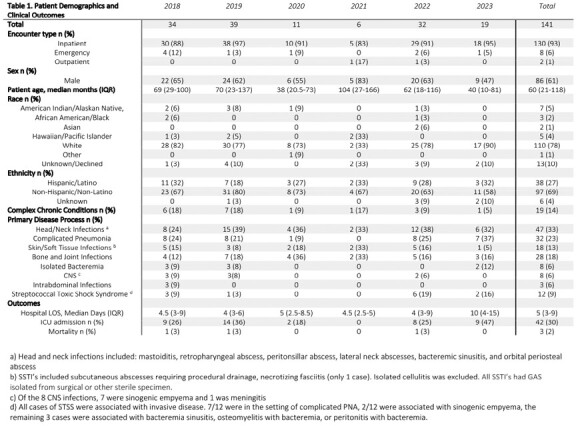

**Figure:**
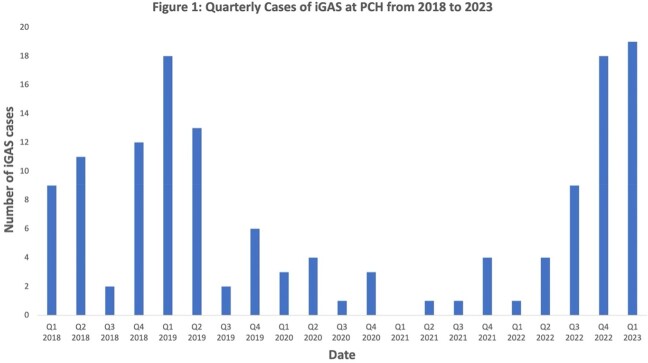

**Conclusion:**

After a marked drop in iGAS infections in children during the COVID-19 pandemic in Utah there was a resurgence of iGAS starting in late 2022 and early 2023, modestly exceeding pre-pandemic levels. *emm1* and *emm12* predominated in the surge. Further analysis of disease severity and molecular epidemiology are in progress.

**Disclosures:**

**Kwabena Krow Ampofo, MD**, Merck: Advisor/Consultant|Merck: Grant/Research Support **Andrew T. Pavia, MD**, GlaxoSmith Kline: Advisor/Consultant|Sanofi: Advisor/Consultant **Anne J. Blaschke, MD, PhD**, BioFire Diagnostics: Grant/Research Support|BioFire Diagnostics: I have IP owned by the U. of Utah licensed to BioFire and receive royalties. I have been a consultant and received grant support as well.|Merck and Company, Inc.: Advisor/Consultant

